# The gut microbiota: an emerging modulator of drug resistance in hepatocellular carcinoma

**DOI:** 10.1080/19490976.2025.2473504

**Published:** 2025-03-05

**Authors:** Jiali Yao, Beifang Ning, Jin Ding

**Affiliations:** a Clinical Cancer Institute, Center for Translational Medicine, Naval Medical University, Shanghai, China; b Department of Gastroenterology, Changzheng Hospital, Naval Medical University, Shanghai, China

**Keywords:** Gut microbiota, drug resistance, hepatocellular carcinoma

## Abstract

Liver cancer is usually diagnosed at an advanced stage and is the third most common cause of cancer-related death worldwide. In addition to the lack of effective treatment options, resistance to therapeutic drugs is a major clinical challenge. The gut microbiota has recently been recognized as one of the key factors regulating host health. The microbiota and its metabolites can directly or indirectly regulate gene expression in the liver, leading to gut–liver axis dysregulation, which is closely related to liver cancer occurrence and the treatment response. Gut microbiota disturbance may participate in tumor progression and drug resistance through metabolite production, gene transfer, immune regulation, and other mechanisms. However, systematic reviews on the role of the gut microbiota in drug resistance in liver cancer are lacking. Herein, we review the relationships between the gut microbiota and the occurrence and drug resistance of hepatocellular carcinoma, summarize the emerging mechanisms underlying gut microbiota-mediated drug resistance, and propose new personalized treatment options to overcome this resistance.

## Introduction

To date, liver cancer remains one of the deadliest and most challenging malignancies to treat effectively worldwide. Despite recent therapeutic advances, the 5-year survival rate of liver cancer remains low.^1^ A major factor underlying the dismal prognosis is the nearly universal development of drug resistance.^2^ Hepatocellular carcinoma (HCC) is the most common pathological type of primary liver cancer and accounts for ~90% of cases. Therefore, this review focuses on the drug resistance in HCC. HCC cells acquire a multitude of genetic and epigenetic alterations that allow them to evade the cytotoxic effects of chemotherapy, posing a major obstacle to improving patient outcomes. The most widely used first-line agent for advanced HCC treatment is sorafenib. However, the objective response rate to sorafenib monotherapy is only 2–3%, with most patients developing acquired resistance within 6–8 months.^3^ Novel molecular targeted drugs such as lenvatinib and regorafenib provide marginal benefits over sorafenib, underscoring the pressing need for combination regimens to overcome resistance.^4,5^ In addition to chemotherapy, immune checkpoint inhibitors, a modality of cancer immunotherapy, have reshaped the therapeutic prospects for multiple types of tumors. Unfortunately, single-agent immunotherapy has demonstrated only modest therapeutic efficacy in HCC, with response rates ranging from 10–20%.^6^ This lack of efficacy highlights the existence of primary resistance pathways that blunt antitumor immune responses in the HCC microenvironment.^7^ Dual immunotherapy strategies are now being explored for their ability to improve treatment efficacy, although complete and durable responses remain uncommon.^8^ A clearer understanding of the precise molecular alterations driving chemoresistance and immune resistance in HCC is imperative for guiding the development of rational combination therapies.

The gut microbiota is composed of bacteria, viruses, fungi and other microbes and is the most densely populated microbial community in the body.^9^ Alterations in the gut microbiome, known as dysbiosis, have been linked to tumorigenesis and tumor drug resistance. Microbial mechanisms associated with tumorigenesis include the production of toxic metabolites, genotoxin-induced DNA damage, the activation of cell proliferation signaling, and the establishment of an inflammatory microenvironment.^10^ Specific bacterial pathogens, such as *Helicobacter hepaticus* and *Fusobacterium nucleatum*, have been associated with HCC.^11,12^ Moreover, fecal microbiota transplantation (FMT) from cancer patients into germ-free mice was found to promote hepatic tumor growth, confirming the role of intestinal dysbiosis in hepatocarcinogenesis.^13^ In addition to contributing to tumor development, the gut microbiota has been recognized as a crucial variable influencing the response to cancer pharmacotherapy. Microbial enzymes can directly metabolize drugs in the intestinal lumen, altering their bioavailability and efficacy.^14^ Bacterial products and metabolites also modulate the expression of genes involved in drug activation, inactivation, and elimination.^15^ Furthermore, the microbiome impacts host signaling pathways that are dysregulated in malignancies as well as the antitumor immune response.

In summary, emerging evidence over the past decade suggests that the gut microbiota may critically influence the response to cancer treatment and the development of drug resistance.^16^ The gut microbiota interacts dynamically with the host through metabolic, immune and homeostatic pathways. Microbial products such as short-chain fatty acids provide energy to intestinal cells, whereas bacterial enzymes modify compounds involved in key physiological processes.^17^ The gut microbiome also regulates the maturation and function of the host immune system. Dysbiosis can disrupt this symbiotic relationship to promote chronic inflammation, one of the hallmarks of carcinogenesis.^18^ Consequently, deciphering the mechanisms of microbiota-mediated resistance may aid in the development of novel combination therapies and the discovery of predictive biomarkers to improve the prognosis of liver cancer.

## Impact of gut microbial dysbiosis on HCC progression and the treatment response

### Composition of the microbiota in HCC patients

The human gut microbiota is increasingly recognized as an important factor influencing hepatocarcinogenesis. With the advent of next-generation sequencing, microbiome profiles associated with HCC are beginning to be identified. Several studies have delineated characteristic features of microbial dysbiosis in patients across the spectrum of liver disease.

In patients with chronic hepatitis B virus (HBV) infection, the gut microbiota undergoes progressive changes during the transition from chronic hepatitis B to cirrhosis to HCC.^19^ Researchers used internal transcribed spacer 2 (ITS2) rDNA sequencing to analyze gut microbiota in stool samples from HCC patients and healthy people and found that, compared with those from control individuals, the relative abundance of beneficial bacterial genera such as *Faecalibacterium* was decreased and the relative abundance of opportunistic pathogens such as *Escherichia/Shigella* was increased in HCC patients. Reduced microbial diversity is also more strongly associated with HCC than with chronic hepatitis B alone.^20^ Similar trends have been found for hepatitis C virus (HCV) infection, with decrease in the relative abundance of butyrate-producing bacteria from the order *Clostridiales* observed in patients with HCV-related HCC.^21^ *Fusobacterium spp*. are also enriched in HCV-infected patients with HCC compared with those with cirrhosis, and this enrichment is correlated with poorer outcomes.^22^ In one study, a large number of stool samples were collected from patients with HCC, patients with cirrhosis, and healthy controls from different regions, and sequencing was performed with the MiSeq system to analyze the gut microbiome. The data revealed that fecal microbial diversity increased during the progression of cirrhosis to early HCC. The abundance of *Actinobacteria* was increased in early HCC patients compared with cirrhosis patients. Similarly, 13 genera, including *Gemmiger* and *Parabacteroides*, were enriched in early HCC patients compared with cirrhosis patients.^23^ This study is a landmark study, which not only provides the first characterization of the gut microbiome of HCC patients, but also establishes a diagnostic model for HCC using microbial markers.

Gut dysbiosis is pronounced in metabolic dysfunction-associated steatotic liver disease (MASLD) and its advanced form, metabolic dysfunction-associated steatohepatitis (MASH), in addition to viral hepatitis and cirrhosis. MASLD and MASH can progress to cirrhosis and HCC if left untreated. The gut microbiomes of patients with MASLD/MASH were found to be characterized by a reduced microbial diversity and the expansion of bacteria populations of the *Enterobacteriaceae* and *Desulfovibrionaceae* families.^24^ Oral administration of stool from MASH mice promoted MASLD progression in germ-free mice compared to that with administration of stool from healthy controls, confirming the direct role of dysbiosis in disease pathogenesis.^25^

Across diverse liver pathologies, several common microbial signatures associated with HCC have been identified. These include overrepresentation of gram-negative bacteria (e.g., *Escherichia*, *Klebsiella*, and *Fusobacterium*), a lower relative abundance of short-chain fatty acid producers (e.g., *Clostridium* clusters), a greater abundance of pathogens (e.g., *Helicobacter*), and lower overall microbial richness/diversity than in non-HCC controls.^26,27^ Characterizing common patterns of dysbiosis linked with neoplastic transformation may enable the identification of microbiome-based biomarkers to identify patients at highest risk of HCC for targeted surveillance and preventative treatment.

### Impact of dysbiosis on gut‒liver axis activity and HCC treatment

The human gut microbiota interacts dynamically with host physiology through metabolic, immune, and homeostatic pathways. Disruption of this symbiotic relationship, a state termed dysbiosis, can have far-reaching effects on liver function and predispose patients to more rapid hepatocarcinogenesis. The gut‒liver axis facilitates multidirectional communication among the intestinal microbiota, intestinal barrier, liver parenchyma, and systemic circulation to maintain hepatic and systemic homeostasis. However, chronic disruptions in signaling via this axis through dietary inputs, inflammation, and other factors can lead to MASLD, facilitating HCC development.^28^ Elucidating the mechanisms by which dysregulation of gut‒liver axis signaling promotes carcinogenesis is key for the development of targeted prevention and treatment strategies. A “leaky gut” is a central contributor to many pathological changes in the gut‒liver axis that enable the development of MASLD and HCC. Impaired intestinal barrier integrity allows the translocation of bacteria, endotoxins, and toxic metabolites into the portal circulation.^29^ Lipopolysaccharide (LPS), a component of the outer membrane of gram-negative bacteria triggers Toll-like receptor 4 signaling in Kupffer cells to initiate hepatic inflammation through NF-kB activation and cytokine release. Secondary effects include dyslipidemia and insulin resistance, which further drive MASLD progression.^30^

Changes in intestinal permeability arise from broad shifts in the microbial composition, depletion of beneficial bacterial species, and weakening of tight junctions due to inflammatory mediators.^31^ Gut dysbiosis and small intestinal bacterial overgrowth are closely linked with increased intestinal permeability in patients across the MASLD spectrum.^32^ A reduced abundance of bacteria, such as *Faecalibacterium prausnitzii*, that increase mucosal integrity occurs in the setting of MASLD.^33^ Microbial metabolites such as short-chain fatty acids nourish enterocytes and maintain tight junctions, underscoring the relevance of the microbial community structure to intestinal barrier function.^34^ In addition to the increased translocation of bacteria and LPS, a leaky gut enables the paracellular uptake of many compounds that promote hepatic inflammation and carcinogenesis. Phosphatidylcholine metabolites such as choline, glycine betaine, and carnitine are depleted in the setting of MASLD, hindering their protective anti-inflammatory effects in the liver.^35^ The transport of toxic bile acids and metabolic byproducts exacerbates oxidative stress and injury. Increased transport of drugs, dietary components, and environmental chemicals to the liver also increases susceptibility to DNA damage, fibrosis, and tumorigenesis.^36^

In addition to affecting gut‒liver axis signaling, the microbial community structure has been reported to influence HCC progression and the response to HCC therapy. Chronic inflammation is a major driver of HCC development, and the gut microbiota critically regulates inflammatory responses. Bacterial products such as LPS activate hepatic macrophages to stimulate NF-kB-mediated cytokine and chemokine production.^37^ The resulting release of inflammatory mediators modulates the tumor microenvironment to promote angiogenesis, cell proliferation, and survival.^38^ Dysbiosis increases the translocation of inflammatory stimuli such as LPS across the intestinal epithelium while also reducing the levels of anti-inflammatory metabolites. Specific microbial pathogens have been mechanistically linked with hepatocarcinogenesis in animal models. *H. hepaticus* infection induces persistent hepatic inflammation that progresses to dysplasia and hepatic tumor development.^39^ Enterotoxigenic *Bacteroides fragilis*, which is enriched in some HCC patients, directly damages DNA through the activity of its toxins.^11^ The oral pathobiont *Porphyromonas gingivalis* accelerates MASLD and MASH in mice, likely by increasing LPS-mediated inflammation.^40^ Depleting such procarcinogenic species may slow tumor progression. In addition to its effects on inflammation, the microbiota influences HCC via other mechanisms. Bacterial β-glucuronidase enzymes promote carcinogen deconjugation and reactivation within the intestine.^41^ The production of genotoxic hydrogen sulfide and secondary bile acids, which cause DNA damage, increases with increasing severity of dysbiosis. A decrease in the relative abundance of beneficial microbes such as *Akkermansia spp*. negatively impacts mucosal barrier integrity, facilitating greater toxin absorption.^42^

With respect to HCC treatment, the microbiome also appears to modulate sensitivity to chemotherapy and immunotherapy. Antibiotic-induced dysbiosis was found to reduce oxaliplatin efficacy in HCC models, which was attributed to impaired drug metabolism.^16^ This finding reveals new approaches based on microbiome-targeted interventions to increase the efficacy of chemotherapeutic drugs. A dynamic analysis of HCC patients receiving anti-PD-1 immunotherapy revealed significant differences in the β diversity of gut microbes between immune responders and nonresponders, with fecal samples from immunoresponsive patients showing greater taxonomic richness and higher gene counts. With immunotherapy, the microbial composition in the responders, which consisted mainly of *Myxomycetes* and *Ruminococci*, remained relatively stable. *Proteus* have also been found to become dominant with treatment progression. These findings suggest that the dynamic changes in gut microbial diversity and composition during immunotherapy for HCC may affect drug efficacy and disease prognosis and that monitoring these changes may allow early prediction of the outcome of immunotherapy in patients with HCC.^43^

In summary, intestinal permeability facilitates a cascade of pathological events in the gut‒liver axis that drive MASLD progression and HCC development. Modulating the gut microbiota, reducing bacterial translocation, and stabilizing intestinal tight junctions are promising adjuvant approaches for suppressing chronic hepatic inflammation and associated malignant transformation. Moreover, characterizing specific features of dysbiosis that accelerate HCC progression and treatment failure will inform the rational design of microbiota-based prevention and treatment strategies.

## Multiple mechanisms underlying microbiota-mediated drug resistance in HCC

### Specific microbial species involved in HCC drug resistance

While gut microbial dysbiosis broadly influences hepatocarcinogenesis, key bacterial pathogens are specifically implicated in promoting drug resistance. Selective targeting of these resistance-promoting species is a potential approach to increase drug efficacy. *F. nucleatum*, one of the bacteria associated with colorectal cancer, is an oral anaerobe. It is also enriched in the stool of HCC patients and induces chemoresistance. *F. nucleatum* infection impairs cytotoxic T-cell responses to create an immunosuppressive microenvironment; the adhesin FadA, a virulence factor, activates prosurvival signaling cascades by binding E-cadherin on tumor cells.^44^ Depletion of *F. nucleatum* resensitized colon cancer cells to chemotherapeutics in preclinical models. Similarly, *P. gingivalis* translocates from the oral cavity to the liver, where it accelerates MASLD progression by increasing the levels of TNF-α, Il-1β, galectin-3, and pSmad2; the number of hepatic crown-like structures (HCLSs); and the MASLD activity score.^45^ It upregulates multidrug resistance transporters in hepatic cancer cells while also activating JAK1/AKT/STAT3 signaling to suppress apoptosis.^46,47^ *P. gingivalis* also decreases the ATP-mediated activation of P2X7 receptors on dendritic cells, which impedes the activation of the NLRP3/ASC/caspase-1 inflammasome. *P. gingivalis* inhibits IL-1β secretion and subsequently induces the generation of IFN-γ-producing tumor antigen-specific CD8^+^ T cells.^48^ Additionally, *P. gingivalis* infection increases the number of myeloid-derived suppressor cells, which impedes antitumor immunity. Microbe-derived mithramycin A has demonstrated particular efficacy against this pathogen. The abundances of *Bacteroides fragilis* strains are relatively high in HCC patients, where they induce genotoxicity and interfere with tumor suppressor pathways. *Enterococcus faecalis* produces extracellular superoxide anions and hydroxyl radicals, which contribute to oxidative liver injury. Treatment with antibodies specific for *E. faecalis* superoxide dismutase limits its carcinogenic effects.^49^

The need for targeted antimicrobial interventions is underscored by evidence indicating that broad-spectrum antibiotics can decrease chemotherapeutic efficacy by depleting commensal microbes such as *Lachnospiraceae*. While antibiotics may help to eliminate select resistance-promoting pathogens, they also disrupt colonization resistance and antitumor immunity.^50^ Overall, identifying specific bacterial mediators of drug resistance provides an opportunity to improve the effects of cancer treatment through selective modulation of the microbiome. Table 1 summarizes the microbes associated with HCC and liver disease stages.

**Table 1.** Microbiome constituents associated with drug resistance in HCC and liver disease stages.

Microbe	Change in abundance	Associated liver disease	Effect/mechanism	Ref.
***Helicobacter hepaticus***	↑	Hepatitis; HCC	↑ persistent hepatic inflammation	39
***Bacteroides fragilis***	↓	Intrahepatic cholestasis; Alcohol-associated liver disease; Hepatic abscess	↑ enterotoxin secretion ↑ host cell DNA damage ↑multidrug resistance protein expression	51–54
***Porphyromonas gingivalis***	↑	MASLD; MASH	↑LPS-mediated inflammation ↑TNF-α, Il-1β, galectin-3, pSmad2 levels ↑multidrug resistance transporter expression ↓apoptosis	40,45–47
***Akkermansia***	↓	MASLD; Metabolic dysfunction-associated fatty liver disease	↑ mucosal barrier integrity ↑ dendritic cell recruitment ↑ T-cell activation ↓ toxin absorption	42,55–57
***Fusobacterium nucleatum***	↑	HCC; Liver metastasis of tumor; Hepatic abscess; Liver failure	↑ autophagy pathway activity ↑ chemoresistance ↓ cytotoxic T-cell responses	11,58–60
***Enterococcus faecalis***	↑	Oxidative liver injury; Alcohol-associated liver disease; Liver cirrhosis	↑ extracellular superoxide anion levels ↑ hydroxyl radical levels	49,61,62
***Clostridium***	↓	HCC; MASLD	↑ short-chain fatty acid levels	26,27,56
***Faecalibacterium prausnitzii***	↓	MASLD	↑ mucosal barrier integrity ↑ butyrate-producing bacterial abundance ↑ dendritic cell recruitment ↑ T-cell activation	33,55
***Bifidobacterium pseudocatenulatum***	↓	Liver cirrhosis; Chronic hepatitis	↑immunosuppressive effects ↑anti-PD-1 resistance ↑ anti-inflammatory factor levels	63–65
***Coprococcus***	↓	MASLD; Hepatic encephalopathy	↓intestinal mucus layer damage	66–68
***Escherichia***	↑	MASLD; Liver cirrhosis	↑ endothelial-to-mesenchymal transformation of liver sinusoidal endothelial cells ↑ inflammation ↑ fibrosis ↓ albumin production	69,70
***Streptococcus***	↑	Alcohol-associated liver disease; MASH	↑ inflammation	71,72
***Veillonella***	↑	Autoimmune hepatitis; Alcohol-associated liver disease; HCC; Liver cirrhosis	↑lipopolysaccharide biosynthesis ↑inflammation ↑intestinal epithelial barrier damage	62,73–76

### Effects of the microbiota on chemoresistance in HCC

Multiple lines of evidence indicate that the composition and functional capacity of the gut microbiota modulate chemosensitivity and chemoresistance in patients with HCC. Elucidating the mechanisms of microbiota-mediated drug resistance can reveal potential new approaches for improving treatment efficacy. The existed mechanisms underlying the acquired resistance of sorafenib include the upregulation of drug efflux pumps, the activation of bypass signaling cascades, and the dysregulation of apoptotic pathways.^77^ One recent study reported that HCC patients with an abundance of gut *Firmicutes*, such as *Clostridium spp*., had significantly longer progression-free survival when taking sorafenib than did those with a predominance of *Bacteroidetes*. Metabolites derived from *Firmicutes* appear to increase the efficacy of sorafenib. This increase may be due to the presence of a microbiome that protects against the response of tumors to chemotherapy. In addition, studies have shown that antibiotic-treated mice and germ-free mice exhibit poor responses to chemotherapy, manifested by reduced cytokine production and insufficient production of reactive oxygen species after treatment.^78^ Antibiotic-induced microbiome disruption has also been found to reduce the effectiveness of chemotherapy in HCC models. Oral vancomycin therapy decreases oxaliplatin sensitivity by eliminating gram-positive bacteria, consequently decreasing the exposure of liver tissue to cytoprotective short-chain fatty acids.^16^ Similarly, ciprofloxacin impairs gemcitabine activity by depleting commensals that metabolize the drug into its active forms. These findings reveal how antibiotic use may inadvertently promote chemoresistance through nonspecific effects on the microbiota. Specific pathogens also drive therapeutic resistance in HCC. In one study, combined biosignature analysis and *in vivo/ex vivo* experiments revealed that the oral bacterium *F. nucleatum* activated the autophagy pathway by targeting the TLR4- and MYD88-dependent innate immune signaling pathways and specific microorganisms to induce chemoresistance. Later, 16S rRNA sequencing and real-time polymerase chain reaction (PCR) were used to examine the abundance of *F. nucleatum* in patient tumor tissues, which was found to be positively correlated with the activation status of the autophagy pathway.^11^ This study, via analysis of human clinical tissues by approaches ranging from bioinformatics analysis to animal/cell experiments, comprehensively confirmed the *F. nucleatum*-mediated chemoresistance. These findings are highly important for the future detection and targeting of *F. nucleatum* and its related pathways. Enterotoxigenic *Bacteroides fragilis* increases the expression of multidrug resistance proteins in HCC cells. Secreted *Bacteroides fragilis* toxins can trigger a procarcinogenic multistep inflammatory cascade dependent on IL-17 R, NF-κB, and STAT3 signaling and directly damage host cell DNA.^51^ Strategically targeting this species and other resistance-promoting species may help to overcome their deleterious effects.

**Figure 1.**
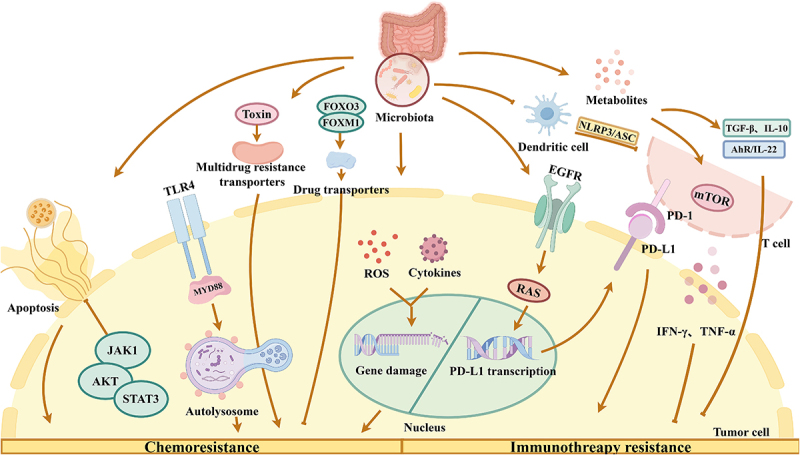
The effect of the microbiota on drug resistance in HCC. The intestinal microbiota can induce drug resistance by inhibiting tumor cell apoptosis through JAK1/AKT/STAT3 signaling, through TLR4/MyD88-induced autophagy, by producing toxins to upregulate multidrug resistance transporters, and through the production of ROS and cytokines that damage DNA in tumor cells. In terms of suppressing chemoresistance, the intestinal microbiota can increase drug carrier sensitivity via the FOXO3-FOXM1 axis. In terms of promoting immunotherapy resistance, the intestinal microbiota can upregulate EGFR and induce drug resistance by promoting PD-L1 transcription and expression through the RAS pathway and by inhibiting the activation of dendritic cells and T cells via NLRP3/ASC. In terms of suppressing immunotherapy resistance, the intestinal microbiota can produce several metabolites that can activate the mTOR signaling pathway to promote the production of IFN-γ and TNF-α by T cells and inhibit drug resistance via AhR/IL-22. Some metabolites can also directly promote TGF-β and IL-10 production to suppress drug resistance.

Additionally, the gut microbiota shapes systemic antitumor immunity, which in turn influences the chemotherapeutic response. A landmark study from Chung’s group revealed, for the first time, the unexpected impact of gut microbes on chemotherapy-induced antitumor immune responses. In their study, 16S rRNA sequencing and quantitative PCR analysis of intestinal mucosa-associated flora in intestinal tissues from cyclophosphamide-treated and untreated mice were performed, and the results revealed that cyclophosphamide can modify the composition of the microbiota and induces the translocation of specific species of gram-positive bacteria to secondary lymphatic organs. These bacteria stimulate the production of a specific subset of “pathogenic” Th17 cells exhibiting antitumor effects and elicit memory T-cell immune responses. Tumor-bearing mice treated with antibiotics targeting gram-positive bacteria exhibited suppression of the immune response. The tumors in these mice were resistant to cyclophosphamide, but adoptive transfer of Th17 cells partially restored the antitumor effects of cyclophosphamide. These results suggest that the gut microbiota influences the antitumor immune response.^50^ Microbiota-derived short-chain fatty acids (such as valerate and butyrate) increase the production of effector molecules such as CD25, IFN-γ and TNF-α by increasing the activity of mTOR, which functions as a central metabolic sensor in cells, and by inhibiting the activity of class I histone deacetylases. Moreover, the infiltration and antitumor activity of antigen-specific cytotoxic T lymphocytes and chimeric antigen receptor (CAR)-T cells are significantly increased.^79^ Overall, integrated modulation of the structure, metabolites, and immune interactions of the microbial community is a promising approach to suppress chemoresistance mechanisms in the context of HCC (Figure 1).

### Interplay between the microbiota and immunotherapy resistance in HCC

The introduction of immune checkpoint inhibitors has reshaped the treatment landscape in multiple tumor types. However, single-agent immunotherapy has demonstrated low efficacy in advanced HCC. The gut microbiota is currently recognized as a key variable influencing antitumor immune responses and the emergence of immunotherapy resistance. Modulation of the microbiome is a promising approach to increase the efficacy of immune checkpoint blockade therapy in HCC patients.

Accumulating evidence highlights the role of the gut microbiota in shaping systemic immunity. Commensal microbes modulate the maturation and activation of dendritic cells, macrophages, granulocytes, innate lymphoid cells and various T-cell subsets.^80^ Metabolites such as short-chain fatty acids can increase the efficacy of PD-1 blockade therapy. This effect is due mainly to their ability to promote the production of anti-inflammatory cytokines and the differentiation of Treg cells, induce a protective immune response and improve epithelial barrier function.^81,82^ Specific species, such as *Akkermansia muciniphila* and *F. prausnitzii*, have been demonstrated to increase dendritic cell recruitment and peripheral T-cell activation.^55^ A eubiotic microbiome promotes optimal priming of tumor-specific immunity. In contrast, dysbiosis is linked to a blunted response to immune checkpoint inhibitors across cancers. Matson *et al*. analyzed the bacteria in fecal samples from patients with metastatic melanoma prior to immunotherapy via 16S rRNA gene sequencing, macrogenomic shotgun sequencing, and quantitative PCR. They found the *bifidobacterium* abundance was positively correlated with the anti-PD-1 efficacy in melanoma patients. Reconstitution of the gut microbiome of germ-free mice by transfer of fecal material from patients responding to therapy led to improved tumor control, augmented T-cell responses, and increased efficacy of anti-PD-1 therapy.^83^ However, some bacteria can activate the expression of the epidermal growth factor receptor (EGFR) by increasing the release of heparin-bound epidermal growth factor (HB-EGF).^84^ Activation of the EGFR/RAS pathway promotes PD-L1 transcription and expression in tumor cells,^85^ which undoubtedly decreases the efficacy of anti-PD-L1 therapy. Antibiotic-induced dysbiosis has been shown to impair immunotherapeutic control in multiple tumor models. One proposed mechanism involves the disruption of the commensal-derived signals needed for cytotoxic T-cell activation and infiltration into the tumor microenvironment. Treatment of mice with *Bacteroides fragilis* or adoptive transfer of *Bacteroides fragilis*-specific T cells effectively overcomes the lack of response to CTLA blockade in antibiotic-treated mice and germ-free mice.^86^ In one study, researchers collected fresh stool samples from HCC patients before and during anti-PD-1 therapy and analyzed the microbiota via macrogenomic sequencing. The differentially enriched taxa revealed an association between gut dysbiosis and innate anti-PD-1 resistance in HCC patients. It was the first study to examine the relationship between the gut microbiome and the clinical response to anti-PD-1 immunotherapy in patients with advanced HCC. Enriched taxa in responders are potential biomarkers for predicting the clinical response and the intervention targets to overcome the immunotherapy resistance. *Bifidobacterium pseudocatenulatum* and *Klebsiella pneumoniae* are selectively enriched in anti-PD-1 nonresponders and may mediate treatment failure through immunosuppressive effects.^63^ In addition, the presence of *Ruminococcus callidus* predicts a good outcome of immunotherapy in many tumors. For example, in patients with HCC, a greater abundance of *R. callidus* is associated with a better response to anti-PD-1 therapy and a higher survival rate. Metabolomic, microbiomic, macrogenomic, and transcriptomic analyses of feces from patients with enterocolitis and control individuals revealed the reduced levels of secondary bile acids and *Ruminococcaceae* in enterocolitis patients. Since the *Ruminococcaceae* family is involved in the production of secondary bile acids, a lack of *Ruminococcaceae* may lead to increased intestinal inflammation and further impacts on the host immune system.^87^ FMT experiments have shown that *Ruminococcaceae* can increase the infiltration of IFN-γ^+^CD8^+^ T cells into tumors to enhance antitumor immunity.^88–90^ Counteracting dysbiosis through modulation of the microbiome could thus help to overcome primary resistance to immunotherapy in HCC patients.

In summary, the microbiota intricately regulates multiple facets of antitumor immunity and represents a promising target for increasing the efficacy of immune checkpoint inhibitors (Figure 1). Defining species that promote or protect against resistance, optimizing synergistic microbe combinations, and identifying predictive microbial biomarkers will be key areas of continued investigation.

### Microbial metabolism and its impact on drug resistance mechanisms

The gut microbiota can directly metabolize and biotransform many pharmaceutical compounds through its vast enzymatic capacity. By modulating the pharmacokinetics and bioavailability of anticancer drugs, microbial metabolism critically influences therapeutic efficacy and resistance pathways in HCC.

Sorafenib is the standard first-line therapeutic agent for advanced HCC. In one study from Edginton’s group, physiologically based pharmacokinetic analyses of sorafenib and its metabolites in mice were conducted, and their data revealed that the gut microbial enzymatic metabolite β-glucuronidase converts sorafenib-glucuronide conjugates into active sorafenib, increasing systemic exposure through enterohepatic recirculation.^91^ However, this increased reabsorption of sorafenib metabolites likely also contributes to its dose-limiting toxicity. Inhibiting microbial β-glucuronidase activity could allow the use of higher sorafenib doses to overcome resistance. Capecitabine is an oral 5-fluorouracil prodrug commonly used to treat gastrointestinal malignancies. Gut microbes metabolize capecitabine into active 5-fluorouracil, and the extent of this metabolism correlates with treatment efficacy.^92,93^ In addition, FOXM1 has been shown to promote HCC progression.^94^ Ghosh *et al*. compared the effects of single-agent (microbial metabolites/5-fluorouracil) and combination (microbial metabolites + 5-fluorouracil) treatment on the chemoresistant HCC cells. In comparison with 5-fluorouracil monotherapy, the combination of 5-fluorouracil with microbial metabolites significantly decreased the viability, proliferation, and invasiveness and induced the apoptosis of drug-resistant tumor cells. Mechanistically, microbial metabolites can sensitize drug transporters and reduce drug efflux by regulating FOXO3-FOXM1 axis signaling, thereby suppressing the progression of chemoresistant tumors.^95^ Butyrate and other short-chain fatty acids induce the expression of key xenobiotic metabolism enzymes that activate capecitabine.^96^ The enrichment of butyrate-producing bacteria such as *F. prausnitzii* could thus increase capecitabine efficacy in tumor patients. In a previous study from Renga’s group, the effect of tryptophan metabolite on the antitumor activity of immune checkpoint blockade was evaluated using a mouse tumor model, and the change in the microbiome induced by tryptophan metabolites was analyzed after FMT. Their results showed that tryptophan metabolites could optimize the therapeutic efficacy of immune checkpoint blockade by affecting the composition and function of the microbiota as well as activating the AhR/IL-22-dependent pathway.^97^ Irinotecan is another prodrug; it is metabolized into the active metabolite SN-38, which undergoes glucuronidation and biliary elimination. Moreover, SN-38 is reactivated by microbial metabolic β-glucuronidases, contributing to dose-limiting diarrhea.^98^ Inhibiting bacterial β-glucuronidase activity could thus allow the use of higher irinotecan doses to overcome resistance in HCC.

In summary, integrated modulation of the microbial community structure, enzyme activity, and metabolites that regulate drug metabolism is a promising approach for suppressing chemoresistance in HCC (Figure 1).

## Therapeutic approaches and strategies

### Potential strategies targeting microbiota to overcome drug resistance

Modulation of the gut microbiome represents a promising approach to increasing the efficacy of chemotherapy and immunotherapy in patients with advanced HCC. Integrated microbiota-targeted strategies aim to deplete resistance-promoting microbes, enrich synergistic commensals, improve metabolic output, and counteract pathological inflammation and immunosuppression. Selectively reducing the relative abundance of pathogens such as *F. nucleatum* that promote chemoresistance is one potential approach.^11^ While broad-spectrum antibiotics disrupt colonization resistance, narrow-spectrum alternatives or bacteriophages could specifically eliminate harmful species. Probiotics such as the *Lactobacillus casei* strain Shirota may impede colonization by opportunistic pathogens.^99^ Ultimately, defining the compositional features of a proinflammatory, proresistance dysbiotic state will help to guide the development of microbiota-restructuring approaches. Prebiotics and synbiotics can nourish beneficial microbes with anti-inflammatory and anticarcinogenic properties, such as *F. prausnitzii* .^100^ FMT is a new direct and effective method for restoring intestinal probiotics and may increase the efficacy of immune checkpoint inhibitors. On the basis of changes in the intestinal lamina propria and gene expression profiles in peritumoral regions, FMT was significantly associated with a transition of the immune system to a state of immune surveillance. FMT is a promising approach for cancer treatment, but it has not been evaluated in clinical trials in HCC patients.^101^ Phages are the natural predators of bacteria, and there are changes in the composition of bacteriophages in the intestinal tract in patients with liver diseases.^62^ Phages that target gut bacteria have been shown to reverse liver disease progression and increase the tumor response to chemotherapy.^102,103^ In addition, the use of prebiotics and antibiotics can destroy harmful bacteria, change the composition of the intestinal microbiota, and improve the microenvironment of the liver.^62,104^ The use of genetically engineered bacteria for targeted therapy is attractive and promising. The specific microbes modified by genetic engineering can not only become a transgenic delivery framework but also regulate the composition, function and metabolites of the local flora.^105–107^ All of these strategies are very important for regulating the drug-resistant microenvironment of HCC.

Targeting microbiota-derived metabolites is another promising strategy to enhance the efficacy of HCC therapy. It has been reported that the enrichment of butyrate producers augments the function of cytotoxic T cells and optimizes the metabolism of specific chemotherapeutic prodrugs.^108,109^ Deaminotyrosine, a microbial metabolite, also promotes T-cell activation and increases the immunotherapeutic efficacy of immune checkpoint inhibitors.^110^ The development of synergistic phytobiotic combinations tailored to overcome unique resistance mechanisms warrants investigation. Inhibiting specific bacterial enzymes, such as β-glucuronidase, may suppress drug metabolism, contributing to resistance.^98^ Moreover, increases in the levels of short-chain fatty acids, secondary bile acids, and other bioactive compounds could synergize with chemotherapy by exerting antiproliferative, proapoptotic, and immunomodulatory effects.^111^

The integrated modulation of microbial community structure, function, and metabolite production through the above-mentioned approaches has the potential to overcome multifaceted resistance mechanisms in HCC. Continued mechanistic clarification of the microbiome’s influence on the therapeutic response and optimization in design of related clinical trials will help to fill the knowledge gaps in these emerging fields.

### Development of microbiota-related therapeutic strategies and future directions

The impact of the gut microbiota in HCC drug resistance and the underlying molecular mechanism are rather complex, but they have attracted tremendous attention in the field. Elucidating the multifaceted roles of the gut microbiome in modulating the therapeutic response in HCC patients requires the integration of new conceptual frameworks, animal models, and omics technologies.

The vast majority of human studies offer only cross-sectional snapshots, obscuring cause‒effect relationships.^27^ Therefore, longitudinal profiling of microbial dynamics from early carcinogenesis through progression and treatment is expected to provide critical insights into the temporal impacts of dysbiosis on drug sensitivity. Models of dextran sodium sulfate-induced colitis and diethylnitrosamine-induced hepatocarcinogenesis can be used to explore changes in the microbiota during inflammation-driven hepatocarcinogenesis.^112^ Genetic engineering and dietary induction models also enable sequential analysis of the tumor microbiome from the initiation stage to the treatment stage.^113^ The use of patient-derived xenografts allows the clinical investigation of paired microbiomes and pharmacologic responses during tumor evolution.^88^ Integrated analysis of pharmacogenomic and microbiomic data may lead to the identification of optimized combinatorial ecopharmacological therapies.^14^

Functional metagenomics moves beyond the microbial composition to characterize the immense metabolic potential of the microbiome. Metagenomic sequencing can identify microbial genes involved in drug activation, inactivation, and efflux that can be targeted to increase drug efficacy.^114^ Metatranscriptomics and metaproteomics illuminate specific drug metabolism pathways that are activated and could be modulated. Integrated analysis of pharmacokinetic and pharmacodynamic data with functional metaomic data provides unparalleled insight into microbe–drug interactions.^14^ Causality testing in gnotobiotic models is the gold standard approach for validating keystone effects of microbes on drug sensitivity. In terms of biological models, the use of germ-free mice colonized with constituents of the microbiota from responders or nonresponders helps to confirm dysbiosis-mediated outcomes, and the transfer of microbiota into conventional mice helps researchers assess the durability of effects.^78^ Defined synthetic communities can be used to delineate interactions between specific commensals. The use of humanized mice colonized with patient-derived microbiota allows *in vivo* analysis of ecological dynamics.^115^ Such capitalization on ecological principles, multiomics datasets, and mechanistic models provides immense potential for revealing the microbiome’s multifaceted impacts on therapeutic resistance in HCC. Continued technological and conceptual innovation promises to foster rapid advancement in this burgeoning field.

**Figure 2.**
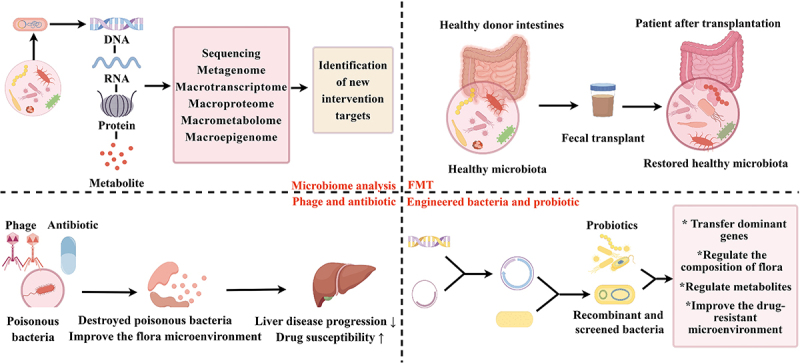
Strategies for targeting the microbiota to overcome drug resistance in HCC. Microbiome analysis methods, such as sequencing, genomic, transcriptomic, proteomic, metabolomic and epigenomic analyses, can improve our understanding of the composition, function, metabolites and epigenetic regulation of the microbiome and subsequently reveal new therapeutic targets. FMT regulates and restores the gut microbiota of the patient via the transfer of bacteria from the stool of a healthy person to the intestines of the patient. Bacteriophages and antibiotics can destroy harmful microflora, improve the microbial microenvironment, inhibit liver disease progression and increase drug sensitivity. The application of engineered bacteria and prebiotics can regulate the composition and metabolite production of intestinal flora and improve the drug-resistant microenvironment.

In parallel, predictive microbial biomarkers are emerging to enable the selection of personalized therapy. Microbiome characterization also aids the monitoring of dysbiosis correction. In summary, the integration of ecological principles, multiomics technologies, and precision approaches facilitates the translation of microbiome research into innovative clinical solutions for overcoming drug resistance. The actualization of rationally designed, microbiome-targeted treatments will usher in a new era in HCC management (Figure 2).

## Challenges and perspectives

In summary, the human gut microbiota has emerged as a critical factor influencing the development, progression, and therapeutic response of HCC. In addition to immune dysregulation and the breakdown of the intestinal epithelial barrier, the type and composition of the intestinal flora are significantly altered in patients with HCC. Some bacteria affect the drug resistance of HCC cells by regulating the local immune response in the tumor environment, activating autophagic signaling, and regulating drug metabolism. Chronic dysbiosis along the gut‒liver axis promotes systemic inflammation,^112^ modulates drug metabolism,^98^ and enables the activation of multidimensional resistance mechanisms that impose major barriers to improving patient outcomes.^11,51^ As research has increasingly focused on the relationship between the gut microbiota and HCC and its treatment, the potential utility of ecology-based integrated approaches that selectively target the microbiota and its metabolites to reduce drug resistance by targeting the regulation of the gut microbiota has been recognized. This realization may lead to the development of a new generation of comprehensive microbiome-oriented treatment strategies.

Given that several technologies have shown therapeutic advantages in HCC patients, there are still some limitations in the existed studies and many challenges in the application of above-mentioned techniques. For example, FMT does not guarantee the accurate transfer of gut microorganisms from donor to recipient, and some beneficial flora may die during the transfer process. Therefore, the development of new techniques for FMT that can maximize the reconstruction of the recipient’s gut microbiota is in urgent need. In addition, after FMT, the recipient’s therapeutic response is influenced by his/her unique gut microbiome or other factors, and does not always parallel the donor’s clinical response. In addition, the classification of responders on the basis of the gut microbiome and the screening criteria for FMT donors should be further explored in future. In terms of probiotic applications, the composition of the gut microbiota is highly complex, and this complexity imposes significant obstacles to the exploration of probiotic‒host interactions in cell culture systems and animal models. Moreover, when targeting microbial metabolites, it is difficult to determine whether the source of the metabolite is the host, the local microbiota or the probiotic. Regarding phages, each phage can target only a limited number of bacteria, and bacteria are likely to develop tolerance when only a single phage is used. Therefore, a strategy incorporating a combination of phages may greatly increase the therapeutic efficacy. Another major problem is the lack of clear reports demonstrating the clinical efficacy of phage therapy for overcoming drug resistance in HCC. In addition, phages are extracted during the bacterial extraction process and contain high levels of endotoxins and other supplements; thus, their safety needs to be noted. Regarding genetically engineered bacteria, questions remain about how to ensure that the engineered bacteria can effectively act on the target flora and cells without harming healthy cells, and how to prevent their clearance by the immune system. More sophisticated methods of targeted drug delivery need to be developed, perhaps by modifying engineered bacteria to recognize specific markers or by designing controlled release mechanisms to deliver therapeutic agents precisely where needed. Encapsulating engineered bacteria in materials that protect them from immune recognition or engineering bacteria to express proteins that can be shielded from the immune system might allow them to escape immune clearance and be more effective. Finally, in terms of exploring the influence of gut microbes on drug resistance in HCC, the depth of research in extant studies is still low. On one hand, most studies focused only on the influence of gut microbes on specific therapeutic effects, and many potential mechanisms underlying drug resistance remain incompletely defined. On the other hand, more preclinical results from animal models and clinical trials are needed to further clarify the efficacy of targeting gut microbes to overcome drug resistance in HCC.

In conclusion, the gut microbiota is undoubtedly a promising new lever to modulate in the bench and clinical research, multiple tumors and modulation of the gut microbiota is among the most promising approaches for overcoming universal therapeutic resistance in patients with advanced HCC.^50,113,116^ Due to the limited studies on the role of microbiota in HCC drug resistance, more efforts should be invested to solve the current problems and fill the existing research gaps.

## Abbreviations

HCC: Hepatocellular carcinoma
ITS2: Internal transcribed spacer 2
HBV: Hepatitis B virus
HCV: Hepatitis C virus
MASLD: Metabolic dysfunction-associated steatotic liver disease
MASH: Metabolic dysfunction-associated steatohepatitis
LPS: Lipopolysaccharide
HCLSs: Hepatic crown-like structures
EGFR: Epidermal growth factor receptor
HB-EGF: Heparin-bound epidermal growth factor
FMT: Fecal microbiota transplantation
